# Highly Insulative PEG-Grafted Cellulose Polyurethane Foams—From Synthesis to Application Properties

**DOI:** 10.3390/ma14216363

**Published:** 2021-10-24

**Authors:** Aleksandra Grząbka-Zasadzińska, Przemysław Bartczak, Sławomir Borysiak

**Affiliations:** Faculty of Chemical Technology, Institute of Chemical Technology and Engineering, Poznan University of Technology, Berdychowo 4, PL-60965 Poznan, Poland; przemyslaw.bartczak@put.poznan.pl (P.B.); slawomir.borysiak@put.poznan.pl (S.B.)

**Keywords:** polymer composites, polyurethanes, nanocellulose, functionalization, rigid foam, physicochemical properties

## Abstract

In this paper, native cellulose I was subjected to alkaline treatment. As a result, cellulose I was transformed to cellulose II and some nanometric particles were formed. Both polymorphic forms of cellulose were modified with poly(ethylene glycol) (PEG) and then used as fillers for polyurethane. Composites were prepared in a one-step process. Cellulosic fillers were characterized in terms of their chemical (Fourier transformation infrared spectroscopy) and supermolecular structure (X-ray diffraction), as well as their particle size. Investigation of composite polyurethane included measurements of density, characteristic processing times of foam formation, compression strength, dimensional stability, water absorption, and thermal conductivity. Much focus was put on the application aspect of the produced insulation polyurethane foams. It was shown that modification of cellulosic filler with poly(ethylene glycol) has a positive influence on formation of polyurethane composites—if modified filler was used, the values of compression strength and density increased, while water sorption and thermal conductivity decreased. Moreover, it was proven that the introduction of cellulosic fillers into the polyurethane matrix does not deteriorate the strength or thermal properties of the foams, and that composites with such fillers have good application potential.

## 1. Introduction

Research on polyurethanes has been carried out continuously for many years. The vast properties of polyurethane (PUR) materials makes them almost irreplaceable in a broad spectrum of, often very sophisticated, applications. Rigid polyurethane foams are commonly used as construction polymers, and thus, the requirements towards them are very high. In today’s world, they refer not only to their physicochemical and mechanical properties but also ecological issues. Recently, particular attention has been paid to ecofriendly, lignocellulosic materials. Numerous types of such materials were already successfully used as fillers for PUR, e.g., peanut shell [1], hazelnut shell [2], tea leaf fiber [3], coconut fiber [4] or potatoes proteins [5]. Nonetheless, cellulose, the main constituent of lignocellulose biomass, seems to attract even more interest. The incorporation of cellulosic fillers to polymer matrix is known to increase mechanical resistance, lower the costs of the final product and affect its biodegradability. Active hydroxyl groups present in the structure of cellulose can form hydrogen bonds, which enable its further modifications [6]. This kind of treatment plays a significant role in the preparation of polymer composites with good dispersion of the filler, increased interfacial adhesion, and thus, better mechanical properties. 

Silva et al. [7] worked on the introduction of cellulose-derived fibers from blanched eucalyptus pulp to polyurethane foams. While the filler addition did not affect the mechanical reinforcement heat and thermal stability, at a concentration of 16%, it caused a reduction in the thermal conductivity of the thermal water (by 32%). The obtained material had relatively good heat-insulating properties. It was also found that under humid conditions, these composites are a favorable environment for fungi, and the authors of the above study claim that it may favor their utilization (ecological aspect). However, such susceptibility to fungi may also have a negative impact on application properties, since PUR materials are often used in humid environments.

Kurańska et al. [8] used microcellulose as a filler for polyurethanes in which the polyol component was synthesized from rapeseed oil. Tests showed that the highest possible content is 9% of the volume of the filler because, at higher concentrations, the polyol premix became too viscous. The presence of microcellulose in the composition resulted in an improvement in mechanical properties (compressive strength and Young’s modulus) and a reduction in brittleness. The addition of this biofiller was also found to enhance fire resistance.

There were also attempts to incorporate nanometric sized cellulose into the PUR matrix. Marcovich et al. [9] reported that strong PUR/nanocellulose interaction results from the chemical reaction occurring between the crystals and the isocyanate component. Wang et al. [10] prepared thermoplastic elastomer polyurethane shape memory composites with cellulose nanofibers as fillers. The high modulus of cellulose nanofibers and rapid water uptake of PUR with nanofibers enabled the production of composites with high shape fixing and recovery ratios that could be applied in biomedical fields. Moreover, other composites of waterborne polyurethane with cellulosic nanofibers were found to be suitable for 3D printing in biomedical applications [11]. Urbina et al. [12] focused on the preparation of bacterial nanocellulose/polyurethane nanocomposites. It was shown that high affinity between the hydrophilic bacterial cellulose and water-stable polyurethane resulted in good water-activated shape memory properties of composites.

Leng et al. [13] proposed different approach; they studied the thermal insulating and mechanical properties of polyurethane foam with cellulose nanofibrils. In comparison to the unfilled sample, the specific bending strength, specific tensile strength, and specific compression strength increased. The thermal conductivity decreased from 0.0439 W/m·K to 0.02724 W/m·K.

Apart from well described advantages of cellulose, it has one problematic property—the hydrophilic nature of cellulose causes it to agglomerate [14]. In terms of composite formation, this is an undesirable phenomenon. Numerous surface modifications (with cationic surfactants [15,16], TEMPO oxidation followed by atom transfer radical polymerization [17], etc.) have been performed to enhance the dispersibility of cellulose. Rivera-Armenta et al. [18] used four different cellulose derivatives: cellulose acetate, carboxymethyl cellulose, cellulose sulphate, and trimethylsilyl cellulose to investigate its influence on the structure and properties of polyurethane foams. All of these compounds have free hydroxyl groups and are capable of reacting in the formation process of polyurethane foams. Their introduction into the polymer matrix changed the cell shape for each derivative, and thus, altered the mechanical properties of the composite. Cellulose acetate was also modified with diphenylmethane-4,4′-diisocyanate for adhesive applications of PUR [19] and polyaniline-coated cellulose nanofibrils were used for segmented polyurethanes that exhibited shape memory [20]. In other study, cellulose nanocrystals were functionalized with a silane coupling agent (γ-aminopropyltriethoxysilane) for the preparation of waterborne PUR [21]. In comparison to non-silanized samples, composites with modified nanocellulose were more thermally stable and had higher tensile strength values, resulting from, i.e., their uniform dispersion in the polymer matrix. However, if the filler content was too high, phase separation occurred. Cellulose nanocrystals were also modified with isophorone diisocyanate, which led to better nanoparticle dispersion, improved thermal stability, and significant increases in the tensile strength of PUR materials [22].

On the other hand, it was reported that a fairly simple adsorption of polyethylene glycol (PEG) onto cellulose nanocrystals enhanced its re-dispersity in water [23,24]. In a study conducted by Pal et al. [25], the addition of PEG-modified nanocellulose and reduced graphene oxide efficiently enhanced the mechanical properties of the polylactide films. Kupka et al. [26] prepared PUR composites with TEMPO-oxidized nanocrystalline cellulose grafted with PEG. Poly(ethylene glycol) was present not only in the filler but also in the matrix, since polyurethane consisted of PEG and aliphatic 1,6-diisocyanatohexane. The results indicated an improvement in tensile properties, which was attributed to stiff particle reinforcement and an increase in the glass transition temperature.

One should keep in mind that, in nature, cellulose exists as cellulose I, but, by using alkali, it can be easily transformed into cellulose II [27]. These two different polymorphic forms have differences in terms of the arrangements of their polysaccharide chains, the sizes of their elementary cells, as well as their crystallinity and particle sizes [28]. Thus, as fillers, they may offer completely distinct mechanical or sorption properties [29,30]. Furthermore, the high availability of renewable cellulose and the low costs of its production make this material an interesting alternative to mineral and synthetic fillers for polyurethanes. Moreover, the use of cellulose functionalization reactions may be an effective method for controlling the formation of polyurethanes and, consequently, could determine the physicochemical properties of foams. This possibility was the primary motivation for this research.

As indicated in the analysis of the above-mentioned publications, even though (nano)cellulose was relatively widely used, there are no data in the literature on the application of different polymorphic forms of cellulose as a component of PUR composites. Furthermore, at present, it has not been established whether the alkali treatment of cellulose and the accompanying changes in its structure, as well as its further modification with poly(ethylene glycol), affects the properties of polyurethane composites. Therefore, the main purpose of this work was to analyze the influence of the PEG modification of two polymorphic forms of cellulose on the application properties of insulation polyurethane foams. To the best of the authors’ knowledge, this is the first publication to deal with this subject. Establishing the relationship between the composition and properties of the PUR/cellulose composite is important from end-user’s point of view, and it is likely to help in the design of better, perhaps less expensive, insulating materials.

## 2. Materials and Methods

### 2.1. Materials

Avicel PH101 (Merck, Darmstadt, Germany) was used as a source of cellulose I. Tin(II) 2-ethylhexanoate (Merck) was used as a catalyst during modification with polyethylene glycol, with M_w_ = 1000 (Merck). Pure NaOH (Chempur, Piekary Śląskie, Poland) was used for the preparation of a 16% solution—the mercerizing agent.

Component A was a mixture of polyols with additives, which was composed of the following: a polyol mixture of various lengths of aliphatic chains (up to 60%), flame retardant (tri (2-chloro-1-methylethyl) phosphate) (up to 15%), a low-boiling organic foaming compound with acceptable ODW and ODP indicators (up to 10%), a catalyst (*N*,*N*-dimethylcyclohexylamine) (up to 5%), stabilizers (up to 5%), and water (up to 5%).

Component B was polymeric 4,4-diphenylmethane diisocyanate (PMDI) with the following properties: content of functional groups NCO = 31–32%; viscosity (at 25 °C) = 210 (mPa/s); density = 1.23 (g/cm^3^).

### 2.2. Preparation of Fillers

For the mercerization process, cellulose I (Avicel PH101) was used. Cellulose was added to 16% NaOH solution and stirred for 15 min. The suspension was neutralized to pH ≈ 7, filtered, and then dried at 105 °C for 24 h. The process parameters were chosen so that the mercerization reaction would result not only in conversion of the cellulose to polymorph II, but also in a decrease in particle size, up to the nanometric scale.

Native cellulose and cellulose treated with alkali were modified using polyethylene glycol (PEG). The process was carried out in a round-bottomed flask, where 1.5 g of cellulose was mixed with 25 mL of PEG and 0.2 mL of tin(II) 2-ethylhexanoate catalyst. The modification lasted for 5 h with intensive stirring at 300 rpm and heating at 90 °C. The obtained material was filtered, washed with water and alcohol, and dried at 105 ° C for 24 h. In further parts of this paper, the following sample names will be used: cellulose I (C I), modified cellulose I (mC I), nanocellulose II (NC II), and modified nanocellulose II (mNC II).

### 2.3. Preparation of Cellulose/PUR Composites

**Figure 1:**
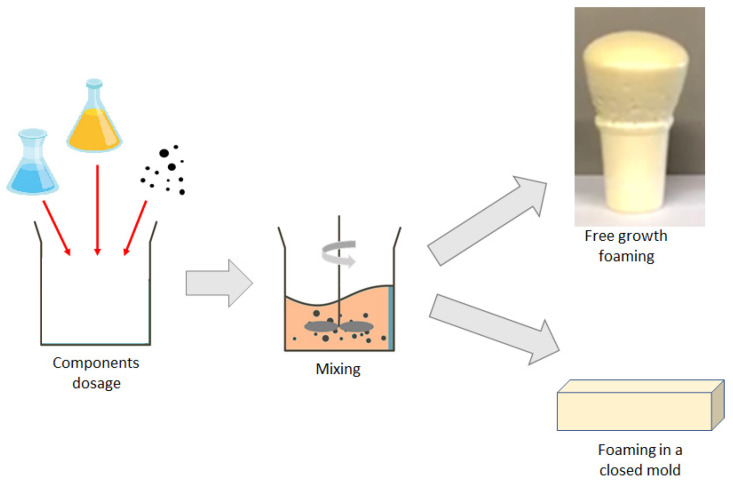
Scheme of composites’ preparation.

PUR composites were prepared using the one-step method, presented schematically in Figure 1.

A quantity of 30 g of the polyol blend (component A) was pre-weighed and the appropriate amount of filler (1 wt.%, 3 wt.% or 5 wt.%) was added. Then, the components were mixed. Finally, 36 g of isocyanate blend (component B) was added and whole system was mixed for 10 s at 3600 rpm. Foaming of samples was performed in two ways: in a cup, allowing free growth of a foam; and in a closed mold. Foam obtained via the molding method was subjected to following types of analysis: water sorption; dimensional stability; thermal conductivity; and compression tests. Cup foamed samples were used for calculations of density and for determining the characteristic processing times.

Composite samples were named so that, i.e., PUR/5% NC II depicted the sample with 5% loading of nanocellulose II. The compositions of the samples are presented in Table 1.

### 2.4. Characterization of Materials

The particle size and the dispersive properties of the cellulosic samples were measured using a Zetasizer Nano ZS (Malvern Instruments Ltd., Malvern, UK). The apparatus operates in range of 0.6–6000 nm, using the non-invasive backscattering technique. Prior to testing, ca. 0.01 g of the tested material was dispersed in 20 cm^3^ of propanol and then ultrasonicated for 20 min.

Cellulosic materials were analyzed by means of X-ray diffraction. CuKα radiation at 30 kV and anode excitation at 25 mA were used. The diffraction patterns were recorded for the angle range of 5–40°. The measurement steps were 0.05° per 3 s.

FTIR spectra were recorded using a Vertex 70 spectrophotometer, manufactured by Bruker (Ettlingen, Germany), with Fourier transform at a range of 4000–600 cm^−1^, at a resolution of 4 cm^−1^, registering 32 scans. Prior to analysis of the fillers, the samples were dried at 105 °C for 24 h, then mixed with KBr (at the following ratio: 200 mg KBr and 2 mg of sample) and finally pressed to form a tablet.

The characteristic times of foam formation—cream, gelling, growth, and tack-free time—were measured directly after the mixing process was stopped.

The cream time is the point at which the volume of the reaction mixture begins to expand, and the polymerization reaction starts. The gelling time is defined as the time when the foam loses its flow properties. At this moment, it is possible to pull the first cross-linked fibers out of the sample. The growth time is measured when the foam reaches its maximum height and stops growing. The tack-free time defines the time needed for the foam to form a dry layer on its surface, at which point the foam is no longer sticky to touch.

The PUR density was determined by weighing the sample using an analytical scale, and determination of its volume was based on the measurement of its dimensions. The density was calculated using Equation (1):

ρ = m/V(1)

where ρ—density (kg/m^3^); m—mass of the sample (kg); V—volume of the sample (m^3^).

The arithmetic mean of seven replicate determinations was taken into consideration in each case.

The tensile properties of the produced composite foams were defined using the Zwick Roell Allround-Line Z020 TEW testing machine (Zwick Roell, Ulm, Germany, in accordance with the DIN EN 826 standard. The samples had a size of 50 × 50 × 50 mm³. During the testing process, each sample was compressed (initial force 250 Pa) at a constant speed (10%/min). The maximum compressive stress at 10% sample deformation was calculated using Equation (2):

R_c_ = F_c_/A_o_ (2)

where R_c_—maximum compressive stress at 10% sample deformation (kPa); F_c_—maximum compressive force (N); Ao—cross-sectional area of the sample (m^2^).

The arithmetic mean of seven replicate determinations was taken into consideration in each case.

The dimensional stability was determined in accordance with the European standard EN 1604 [31]: (A) at a temperature 85 ± 2 °C without a specific humidity; (B) at a temperature of 70 ± 2 °C and a relative humidity of 90 ± 5% for changes to samples that occurred after 48 h. To obtain test data, a Binder KMF 240 climate chamber (Binder, Tuttlingen, Germany), with temperature and humidity control, was used.

The short-term water absorption tests were carried out in accordance with the European Standard ISO 29767 [32]. The water absorption was calculated from Equation (3), as shown below:

A_water_ = (m_1_ − m_0_)/A (3)

where A_water_—water absorption (kg/m^2^); m_0_—mass of the sample before immersion in water (kg); m_1_—mass of the sample after immersion in water (kg); A—surface area of the immersed sample (m^2^).

Assessment of the thermal conductivity was carried out, in accordance with the European standard EN 12667 [33], using the TAURUS TCA 300 heat meter (Netzsch, Selb, Germany). During the test, the direction of the heat was upwards. Measurements were taken at three test temperatures: 10, 30, and 50 °C.

The morphology and microstructure of the obtained composites were analyzed with a Zeiss EVO40 (Oberkochen, Germany) scanning electron microscope at an acceleration voltage of 5 kV, using secondary electron detectors.

## 3. Results and Discussion

### 3.1. Characterization of Fillers

#### 3.1.1. FTIR

**Figure 2:**
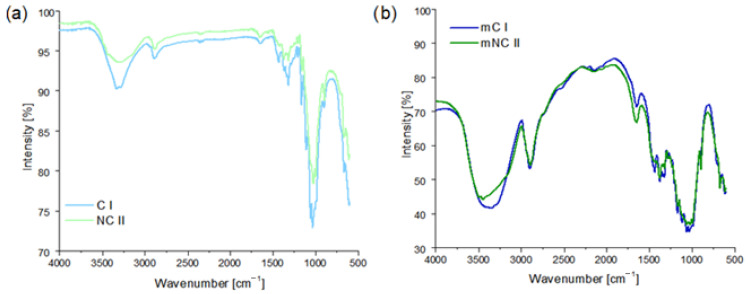
FTIR spectra of native and mercerized cellulose: (**a**) pristine; (**b**) after PEG modification.

Fourier transform infrared spectroscopy was used to identify the characteristic functional groups that were present in the structure of the samples before and after PEG treatment. Figure 2a shows the spectra of the starting celluloses. Both of the spectra exhibited very similar absorption bands, which confirms that mercerization (transformation of C I into NC II) did not alter the chemical structure of the cellulose. In addition, the observed bands are consistent with the characteristic bands reported in the literature (see Table 2) [34].

An infrared study was also carried out on the samples after PEG modification (Figure 2b). When compared to unmodified cellulose I and II, an increase in the intensity and width of the peak was noticed at 3400 cm^−1^. This band corresponds to the stretching vibrations of the –OH groups. In addition, the peaks observed at 2850 cm^−1^, and in the range of 1200–1000 cm^−1^ (assigned to the presence of the –CH and –COH groups, respectively), were more intense that those in Figure 2a. The presence of a band at 1730 cm^−1^ (ester bonds) suggests that PEG was not only absorbed, but also covalently bonded to the surface of the filler. According to the literature [35,36], these changes demonstrate the effectiveness of the PEG modification of celluloses.

#### 3.1.2. Particle Size

Studies on the size distribution of particles were carried out to define the influence of alkaline treatment and PEG modification on the size of cellulose particles.

**Figure 3:**
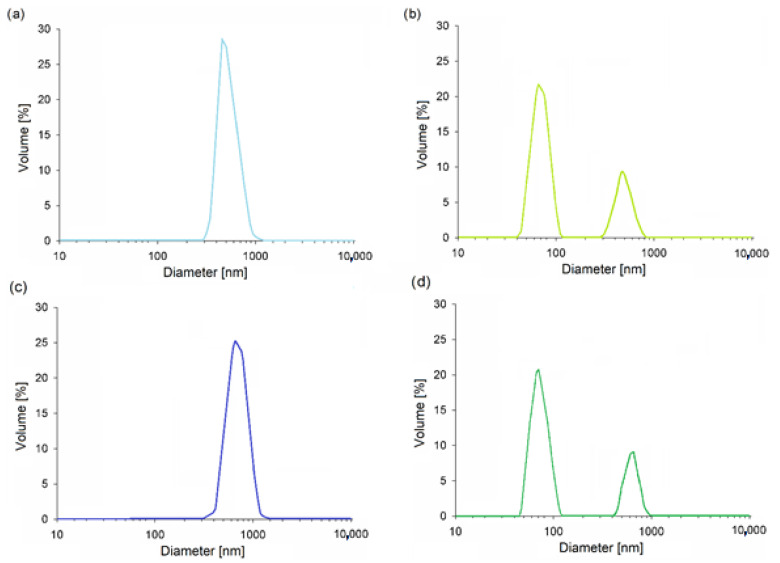
Particle size distribution for: (**a**) C I; (**b**) NC II; (**c**) mC I; and (**d**) mNC II.

The mean particle size for cellulose I was ~50 µm. However, it turned out that the mercerization process caused a decrease of ~40 µm in the particle size. More importantly, a fraction of nanometric particles (50–100 nm), which was not present in starting material, was formed (Figure 3a,b). Therefore, the decision to name the cellulose sample obtained after mercerization as “nanocellulose” is justified. The decrease in cellulose particle size after NaOH can be ascribed to the contraction that occurred during mercerization. It was previously established that this is caused by changes in the structure and orientation of the cellulose molecular chains [37,38]. Moreover, alkali treatment is known to be responsible for the partial depolymerization and shortening of cellulose chains [39].

The results obtained for mC I and mNC II (shown in Figure 3c,d) are very similar to those of the unmodified celluloses. Modification with PEG caused some slight increases in particle size (~60 µm for mC I; 60–130 nm and ~60 µm for mNC II). The main relationship remained unchanged—unlike native cellulose, the sample had two fractions of particles after mercerization: micro- and nanometric. Grafting a high-molecular-weight PEG may cause a tangling effect, leading to an increase in particle size [23]. The data presented in this study are comparable with the results reported elsewhere [26,40].

The fact that alkali treatment of native cellulose can result in the formation of a nanometric fraction has not yet been reported in the literature.

In order to confirm that this modification was also effective in terms of polymorphic conversion, XRD studies were performed.

#### 3.1.3. XRD

**Figure 4:**
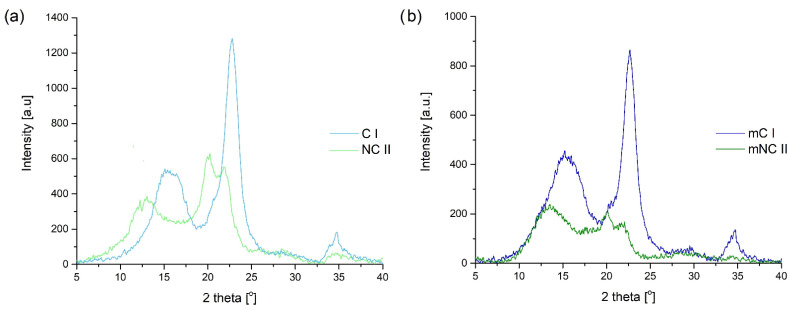
WAXS patterns for native and alkali-treated cellulose: (**a**) before PEG modification; (**b**) after PEG modification.

**Figure 5:**
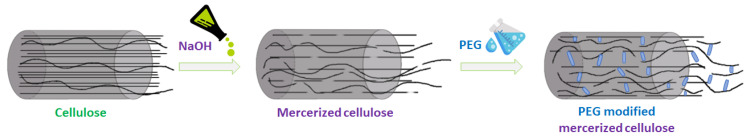
Scheme of modification of cellulose particles.

The effectiveness of alkali treatment of native cellulose was fully confirmed using the XRD method. As shown in Figure 4a, the diffraction maxima for the sample of native cellulose occurred at 2Ө = 15°, 16.5°, 22.5°, and 34.5°. These values are characteristic of cellulose I. After the mercerization process, the locations of the maxima were changed to 2Ө = 12.5°, 20°, and 22°. These observations are in line with the data given in the literature and prove that a change in the crystalline structure of the filler occurred [41,42]. Similar diffraction patterns were recorded for samples after the PEG modification process (Figure 4b); the locations of the peaks for each polymorphic form of cellulose remained the same. However, after grafting with PEG, the intensity of the peaks was decreased. The intensity of diffraction maxima is associated with many aspects, not only the composition but also the morphology of the sample. The higher the number of well-defined crystalline planes, the higher the intensity of the corresponding maxima. Moreover, in comparison to the starting materials, the degree of crystallinity of the modified samples was lower. This suggests that the introduction of the modifier into the structure of the celluloses, especially due to the presence of a steric hindrance of PEG, reduced their ability to crystallize. The decrease in the degree of crystallinity associated with PEG modification was definitely more visible for micrometric cellulose I than for nanometric cellulose II (crystallinity decreased from 72% to 50% for C I and mC I, and from 43% to 38% for NC II and mNC II). The incorporation of PEG molecules into the structure of the cellulose caused a loosening of the structure, which resulted from the breaking of the inter- and intramolecular hydrogen bonds of the cellulose. Figure 5 schematically represents the interactions between the cellulose and the PEG.

### 3.2. Characterization of Composites

#### 3.2.1. Characteristic Processing Times of PUR Formation

The first analysis, which was related to the preparation of PUR foam composites with cellulosic fillers, involved the determination of the characteristic times of foam formation. This is an important parameter that allows the determination of not only the temperature reactivity of PUR foams, but also the processing aspect. For this purpose, the following parameters were measured: cream, gelling, growth, and tack-free time (Table 3).

In terms of cream time, there were no drastic changes; therefore, the results can be considered as comparable (27–30 s).

In all composite samples, the gelling time was slightly shortened. For composites with unmodified fillers, it ranged from 125 to 129 s, while for those with modified fillers, it reached 120–125 s. There are no indications that, at this stage of the foam development process, the particle size or content was a decisive factor. This suggests that the increase in the reactivity of the foam was related to the filler type—PEG-modified vs unmodified filler. The difference between composites before and after PEG treatment could have been due to lower crystallinity of the modified samples (72% and 43% for C I and NC II; 50% and 38% after their modification).

The situation changed and became more complex as the foaming process continued. Firstly, as concentration of the filler increased, due to the incorporation of higher amounts of spatial obstacles, the growth time became longer (e.g., 194 s and 199 s for PUR with 1% mC I and 5% mC I, respectively).

Secondly, composites with the nanometric cellulose II obtained slightly higher values compared to micrometric cellulose I. It is likely that during alkali treatment, the surface area of the cellulosic material was developed and, as a result, there were stronger interactions between the filler and the polymeric components of the foam. It is also known that the addition of the filler can strongly affect the viscosity of the formulation, thereby affecting the movement of polymeric chains and slowing down the reaction rate [43]. Even more interestingly, modification of the filler resulted in a shortening of the growth time of the foam (e.g., 226 s vs. 210 s for composite with 5% unmodified and modified NC II).

Presumably, the modification of cellulose decreased the number of hydrogen bonds between its particles, and hence, loosened the 3D structure, enabling more effective movements of the growing PUR chains. This could be also a reason why the gelling times were affected more when modified filler was applied.

For the tack-free times, it was noted that composites with high filler content had lower values of this parameter. Here, there was no strict relationship between the characteristic time and size of the filler (for micrometric cellulose, it was in the range of 279–290 s, while for nanometric cellulose, it was in the range of 281–291 s). In addition, the PEG modification of micrometric cellulose did not affect this parameter. Even so, the shortest tack-free times were observed for PUR with modified nanometric cellulose II. Alkaline treatment, as well as the PEG modification of nanometric cellulose, resulted in decrease in its degree of crystallinity. Lower crystallinity may facilitate the diffusion of long polymeric chains, resulting in a slight acceleration of the final stages of the polymerization process.

#### 3.2.2. Density

**Figure 6:**
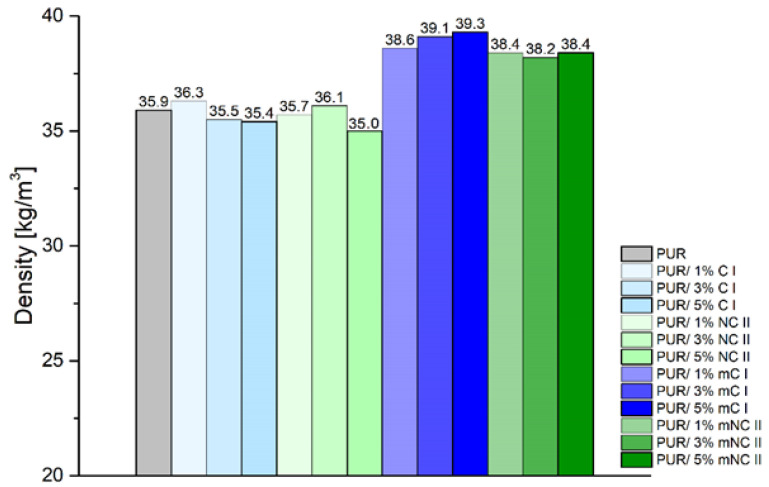
Density of composites.

The use of various types of fillers for rigid foams can affect the free density of the materials obtained. The effect of cellulosic fillers on this parameter is shown in Figure 6.

Composites with C I and NC II filler had a comparable density (35.0–36.3 kg/m^3^) to the reference sample—35.9 kg/m^3^ (Figure 6). Foams to which modified cellulose was added were characterized by higher values, ranging from 38.2 to 39.3 kg/m^3^, wherein minimally (0.5 kg/m^3^) greater density was obtained from the composite with modified cellulose I (mC I). The filler content did not influence the density parameter. In line with the data found in the literature, a similar relationship was demonstrated using cellulose fibers from a blanched mass from eucalyptus [7]. The addition of fibers at a proportion of up to 4% did not change the density of the composite; the difference was noticeable only at levels above 8% of the volume of the filler. The authors of the above study claim that this finding was a result of a decrease in the reactivity of the system, caused by an increase in the viscosity of the mixture, that also affected the expansion of the foam. Moreover, the addition of a filler derived from cloves was found to slightly increase the density of PUR [44]. The obtained density values of the free rigid polyurethane foam correlate well with the other output parameters that are presented in subsequent chapters.

#### 3.2.3. Dimensional Stability

In view of the possible applications of developed composites, dimensional stability was measured, at different temperatures and at constant humidity (85 °C and 70 °C at 90%), only for PUR foams with 5% of the volume of the filler.

The data presented in Table 4 show that the linear changes are similar for all PUR foam composites. An analysis of the dimensional stability of the PUR materials showed no significant differences. Deterioration of the dimensional stability was observed for samples with unmodified fillers because the hydroxyl group in cellulose was still available to react with the surrounding moisture or water. This resulted in the deterioration of the dimensional stability. A slight deterioration in the dimensional stability was obtained for samples with unmodified fillers. In the case of samples with modified cellulose fillers (mC I and mNC II), the obtained stability was at the level of the reference sample.

It is known that some PUR with fillers exhibit dimensional instability and structural damage. This is caused by the reaction of short functional groups, present on the surface of the fillers, with the functional groups of PUR. In this case, the modification of cellulose fillers led to the branching of the polymeric chains and the reaction of free isocyanate groups. This resulted in a compact and stable structure. These results correlate well with the calculated densities shown in the previous subsection. The fact, that the applied fillers did not adversely affect the dimensions of the sample, even though tests were carried out under rigorous conditions, it is a very positive result, showing the application potential of the produced materials.

#### 3.2.4. Water Absorption

The water sorption parameter is extremely important since commercial PUR foam cannot soak up water. The susceptibility to water absorption may intensively affect the thermal insulation and mechanical characteristics of the final PUR product. Therefore, the next step of this study was to determine the water absorption properties.

**Figure 7:**
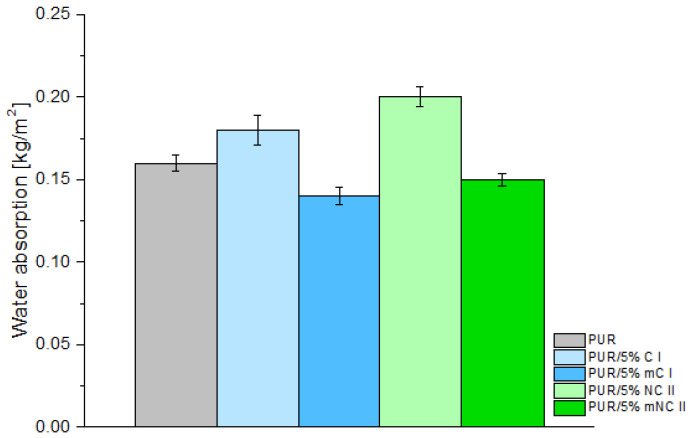
Water absorption of composites depending on filler type (5% loading).

Pristine cellulosic fillers have highly hydrophilic natures, and this is clearly visible in the results of the water sorption test (Figure 7), which showed that the sample with cellulose I and cellulose II absorbed more water than unfilled PUR foam. However, the use of modified cellulosic fillers had a positive effect on this parameter. Polyurethanes with 5% mC I and mNC II were characterized by lower water absorption compared to all other samples, including the reference foam. This is consistent with other papers, in which it is stated that the PEG modification of cellulose decreases its hydrophilicity [23,40]. Higher absorption of water in the PUR with unmodified filler is due to the presence of the hydroxyl groups of cellulose that are still able to react with water. In contrast, the reduction in water absorption in PUR with PEG-modified filler is caused by lower number of hydroxyl groups in the filler. This is a result of the attachment of PEG molecules by means of hydrogen bonds. It may be also assumed that foams prepared with the use of the PEG-modified fillers are characterized by a higher content of closed cells compared to the other materials (with the addition of unmodified cellulose fillers). The presence of closed cells is confirmed by means of the SEM photosshown in the next chapter.

#### 3.2.5. Compression Tests

Compressive strength is another, very important, performance parameter that affects the application of foams.

**Figure 8:**
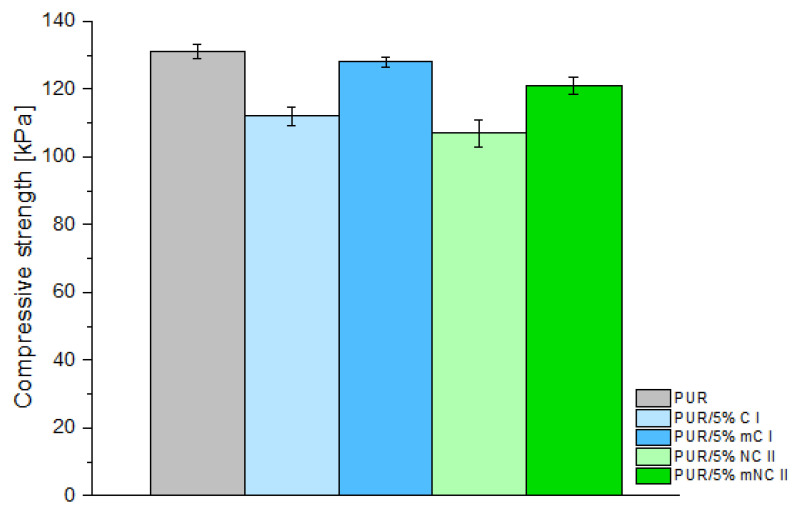
Compression strength of composites depending on filler type (5% loading).

As shown in Figure 8, at 10% deformation of the foams, the maximum compressive forces were above 100 kPa. The highest value, 131 kPa, was noted for the reference sample. The use of unmodified cellulose filler of both polymorphic forms was found to significantly reduce this parameter (112 kPa for the C I filler and 107 kPa for the NC II filler).

If unmodified fillers were used, an increase in the free density of the obtained composites was noted (see Figure 7). This contributed to a greater expansion of the foam and an increase in the cell size. Most likely, the walls of the cells became thinner and weaker, and therefore, the obtained compressive strength parameters were lower. Additionally, the reduction in compressive strength of PUR with unmodified cellulose indicates that not all filler-derived hydroxyl groups were cross-linked with PUR-derived functional groups. It is probable that the accessible hydroxyl groups of the filler were not fully linked and embedded in the PUR structure.

Nonetheless, if PEG-modified fillers were used, there was not much difference in the compressive strength (128 kPa and 121 kPa for PUR with mC I and mNC II, respectively). The obtained free densities of the composites confirm this relationship. For the mC I and mNC II samples, these values were similar to the reference sample and amounted to 35.4 and 35.0 kg/m^3^, respectively. The foam expansion and cell size were similar to the pure PUR sample. Therefore, the composites with modified fillers had similar compressive strength values; there was not much difference between the mC I and mNC II samples.

Below, we present a short literature review showing that the introduction of natural fillers into the structure of PUR foams is known to result in similar dependences of compressive strength.

Członka et al. [45] reported that the addition of 5% walnut shell to polyurethane reduces this parameter by ca. 11%. Silva et al. [7] investigated rigid foams with cellulose fibers. They concluded that, when compared to unfilled sample, the decrease in compressive strength for these composites was not greater than 7%, which is very similar to our result. Such decrease in the value of compressive strength is not significant in terms of the commercial applications of PUR foams.

In our study, the main aspect influencing the discussed parameter was the PEG modification of the filler. The interaction between the filler and the polymer matrix plays a key role in improving the mechanical properties of the foam [46]. The incorporation of (nano)cellulose grafted with PEG enhanced the interfacial adhesion. Moreover, the reduced hydrophilicity of the filler had a positive effect on the uniformity of its distribution in the polymer matrix.

Similar results were obtained by other scientists researching polyurethanes with the addition of cloves [44] and coconut fibers covered with henna [47]. It was found that overly high filler contents in the polymer matrix disturb the homogeneous structure of the material, and thus, make it more prone to mechanical stress. The deterioration may also be due to the agglomeration of filler particles, resulting in poorer adhesion to the matrix.

#### 3.2.6. Thermal Conductivity

**Figure 9:**
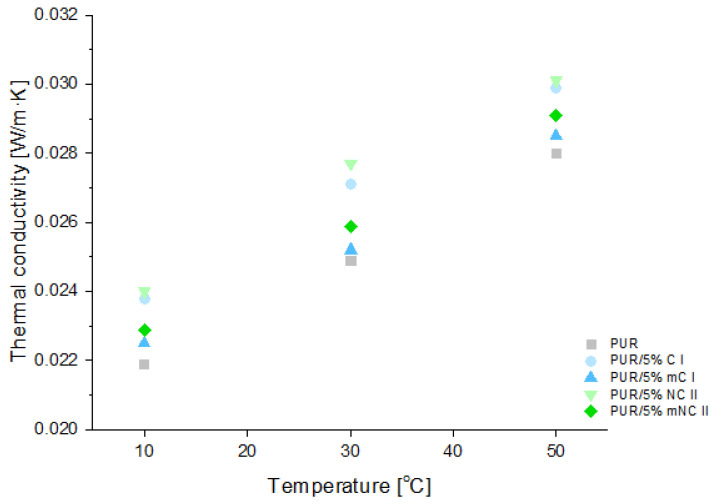
Thermal conductivity of composites depending on filler type (5% loading).

Rigid polyurethane foams are used as thermal insulation materials; therefore, one of their most important parameters is thermal conductivity [48]. The basic parameter for the assessment of these properties is the thermal conductivity coefficient (the λ parameter). Figure 9 presents the values of this parameter for PUR and its composites.

It is worth emphasizing that the thermal conductivity of polyurethane materials depends closely on the porosity of their structure. Therefore, it is extremely important to keep in mind that introducing fillers into the polyurethane matrix may have an influence on the size and shape of the pores. Given that information, it was to be expected that the introduction of fillers to the polyurethane matrix would have some influence on the thermal conductivity coefficient. Even so, the increase in the lambda parameter was very slight (maximum ca. 10% in comparison to the reference PUR value). Interestingly, for composites with modified celluloses, this difference was less marked. For example, at 30 °C, the lambda parameter for the PUR/5 C I sample was 0.0271 W/m·K, and it was 0.0252 W/m·K for PUR/5 mC I (reference sample 0.0249 W/m·K).

However, the data in the literature indicate a significant deterioration of the insulating properties of polyurethane foams containing lignocellulosic fillers. On the other hand, the addition of a 5 wt% volume of salvia filler to the PUR matrix was responsible for the increase in the thermal conductivity from 0.025 W/m·K to 0.035 W/m·K [49]. In addition, the PUR composites that were reinforced with a 20 wt% volume of date palm particles had thermal conductivity values of 0.0389 W/m·K [50]. It was reported in the literature that properties of PUR/natural fillers composites are highly affected by the size and distribution of filler particles. Leszczyńska et al. [51] showed that composites with nut shell were characterized by increased structure anisotropy and higher contents of the irregularly shaped pores than those of the reference sample. Lu et al. [52] investigated PUR samples with up to a 20 wt% volume of wood or lignin. The usage of each filler resulted in the formation of poorer cellular structures, with large numbers of open cells, affecting the final properties of the composite. On the other hand, Tao et al. [53] prepared PUR foams with straw fiber and wheat straw fiber. As the fiber content increased (5, 10, 15, and 20 php), the cell size became smaller and less uniform. In some cases, closed cell structures were even destroyed. Such negative influence of lignocellulosic fillers on polyurethane cell formation were ascribed to the filler particles attaching to the cell, weakening its structure, and finally leading to its destruction [54].

**Figure 10:**
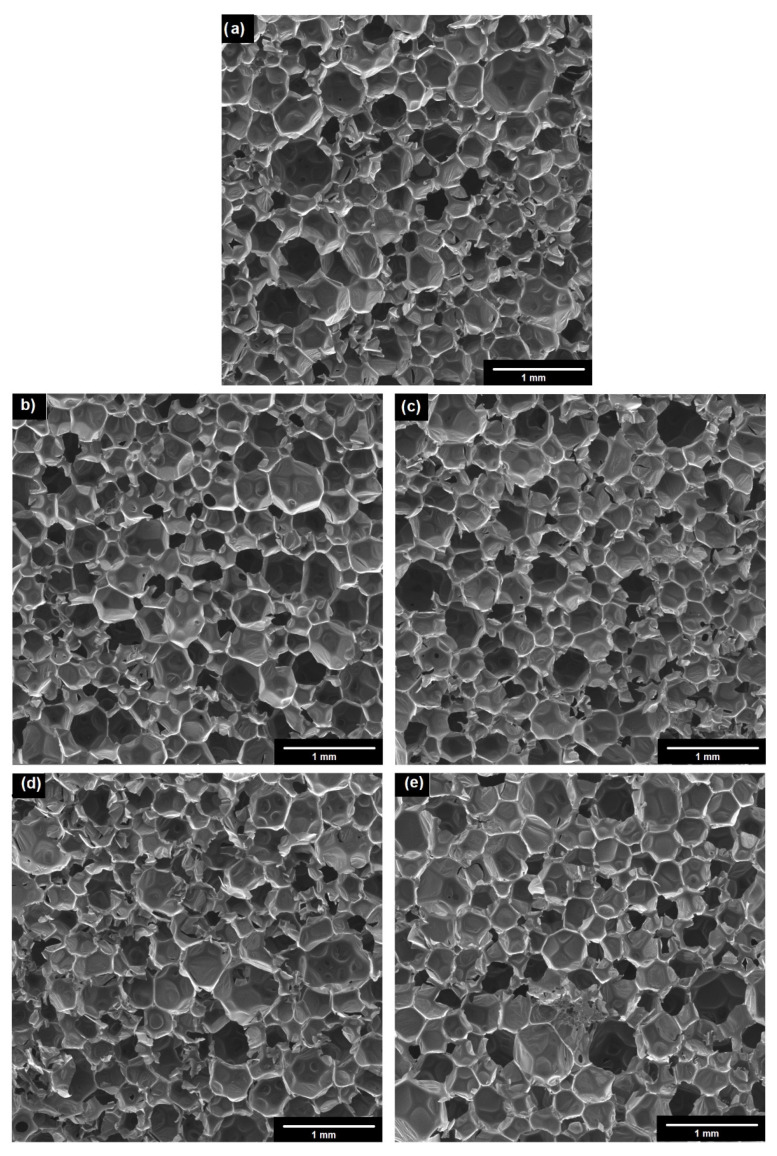
SEM photos of: (**a**) PUR; (**b**) PUR/5% C I; (**c**) PUR/5% mC I; (**d**) PUR/5% NC II; (**e**) PUR/5% mNC II.

SEM pictures (Figure 10) were taken to illustrate the differences in the lambda parameter. Based on the SEM photos, it can be concluded that the obtained foam composites have a microstructure with a significant content of closed cells, which is typical for insulating foams.

In comparison with the reference sample, the composites with unmodified fillers present some changes in their structures and increases in their numbers of open cells. This had a direct influence on the deterioration of the insulating properties, which is confirmed by the results presented in Figure 9 (an increase in the value of thermal conductivity within the whole temperature range indicates a deterioration of the insulation parameters). On the other hand, all PUR composites with modified fillers had similar cell sizes and numbers of closed cells, which is in line with the calculated λ values. This relationship may have resulted from the fact that the filler has the ability to limit the expansion (growth) of cells and, thus, the entire foam structure (this fact is confirmed by the higher densities obtained for these composites—Figure 6).

In general, the λ value of rigid polyurethane foams used as insulation materials should be in range of 0.022–0.030 W/m·K.

In this experiment, none of the obtained polyurethane composites exceeded the required range. Therefore, it should be stressed that in comparison with the results presented in other papers, the ones obtained in this study are still relatively low and do not impair possible applications of the composites.

The best insulating properties were demonstrated by systems that contained modified celluloses. In general, increases in foam density reduce thermal conductivity. Samples with mC I and mNC II had higher density than composites with unmodified fillers, so it is understandable that they performed better.

This better performance could be due to the addition of a modified filler to the PUR matrix. PEG acted as a branching agent, contributing to the creation of the more uniform structure of the composites than in samples that were filled with unmodified fillers. Another aspect could be the number of the closed cells. Structures with high amounts of open cells are characterized by increased heat transfer [49].

## 4. Conclusions

In this study, native cellulose was used as a starting material for the preparation of fillers. In the first step, it was alkali treated. It turned out that the mercerization of cellulose resulted not only in a change in the supermolecular structure (conversion into the polymorphic form that is characteristic of cellulose II) but also a decrease in the particle size.

Measurements of the particle size distribution showed that apart from micrometric fraction, some fraction of nanometric particles (not present in sample of native cellulose) was obtained.

In the next step, both cellulosic fillers were successfully modified with poly(ethylene glycol). All four types of fillers were combined with polyurethan, and thus, composite foams containing 1 wt%, 3 wt%, and 5 wt% volumes of cellulose were obtained. Modified nanometric cellulose II was found to have a catalytic effect on the total foaming time. It was also shown that the modification of celluloses with PEG was effective in terms of producing composites with relatively greater flexibility, increased density, reduced water sorption and lower thermal conductivity than in the case of using unmodified fillers.

Most importantly, it was proven that the prepared composites were characterized by thermal conductivity, density, water absorption, dimensional stability, and compressive strength at a level that is correct and desirable from the practical and technological points of view.

## Figures and Tables

**Table 1:** Compositions of the samples.

Sample Name	Filler Content (%)			
	Cellulose I	Nanocellulose II	PEG-Modified Cellulose I	PEG-Modified Nanocellulose II
PUR	0	-	-	-
PUR/1 C I	1	-	-	-
PUR/3 C I	3	-	-	-
PUR/5 C I	5	-	-	-
PUR/1 NC II	-	1	-	-
PUR/3 NC II	-	3	-	-
PUR/5 NC II	-	5	-	-
PUR/1 mC I	-	-	1	-
PUR/3 mC I	-	-	3	-
PUR/5 mC I	-	-	5	-
PUR/1 mNC II	-	-	-	1
PUR/3 mNC II	-	-	-	3
PUR/5 mNC II	-	-	-	5

**Table 2:** Characteristic FTIR bands.

Wavenumber (cm^−1^)	Band Assignment
3340	O–H stretching
2900	C–H stretching
1730	C–O stretching
1650	O–H stretching
1430	C–H bending
1380	
1330	
1200–1000	C–OH stretching
990	C–O–C stretching
895	C5 and C6 vibrations
650	C–OH stretching

**Table 3:** Characteristic processing times for PUR and its composites.

	Cream Time (s)	Gelling Time (s)	Growth Time (s)	Tack-Free Time (s)
PUR	29	131	185	280
PUR/1% C I	30	129	204	290
PUR/3% C I	30	128	209	284
PUR/5% C I	27	125	207	279
PUR/1% NC II	29	129	199	291
PUR/3% NC II	28	125	216	287
PUR/5% NC II	27	126	226	281
PUR/1% mC I	27	120	194	290
PUR/3% mC I	27	124	196	280
PUR/5% mC I	28	125	199	278
PUR/1% mNC II	27	122	197	276
PUR/3% mNC II	28	121	205	271
PUR/5% mNC II	28	120	210	269

**Table 4:** Dimensional stability of composites depending on filler type.

Sample	Dimensional Stability at 85 ± 2 °C without a Specific Humidity (%) ± 1			Dimensional Stability at 70 ± 2 °C and Relative Humidity 90 ± 5% (%) ± 1		
	Length	Width	Thickness	Length	Width	Thickness
PUR	0.5	0.5	0.6	0.9	0.8	0.8
PUR/5% C I	0.8	0.7	0.7	1.2	1.3	1.0
PUR/5% mC I	0.6	0.6	0.5	1.0	0.9	0.7
PUR/5% NC II	0.9	0.9	0.8	1.2	1.4	1.1
PUR/5% mNC II	0.6	0.8	0.6	1.1	1.1	0.9

## Data Availability

Data available on request.
